# Expression of SET Protein in the Ovaries of Patients with Polycystic Ovary Syndrome

**DOI:** 10.1155/2013/367956

**Published:** 2013-06-03

**Authors:** Xu Boqun, Dai Xiaonan, Cui YuGui, Gao Lingling, Dai Xue, Chao Gao, Diao Feiyang, Liu Jiayin, Li Gao, Mei Li, Yuan Zhang, Xiang Ma

**Affiliations:** ^1^The State Laboratory of Reproductive Medicine, Clinical Center of Reproductive Medicine, First Affiliated Hospital, Nanjing Medical University, Nanjing 210029, China; ^2^Department of Obstetrics and Gynecology, Second Affiliated Hospital of Nanjing Medical University, Nanjing 210011, China; ^3^The First Affiliated Hospital, Nanjing Medical University, China

## Abstract

*Background*. We previously found that expression of SET gene was up-regulated in polycystic ovaries by using microarray. It suggested that SET may be an attractive candidate regulator involved in the pathophysiology of polycystic ovary syndrome (PCOS). In this study, expression and cellular localization of SET protein were investigated in human polycystic and normal ovaries. *Method*. Ovarian tissues, six normal ovaries and six polycystic ovaries, were collected during transsexual operation and surgical treatment with the signed consent form. The cellular localization of SET protein was observed by immunohistochemistry. The expression levels of SET protein were analyzed by Western Blot. *Result*. SET protein was expressed predominantly in the theca cells and oocytes of human ovarian follicles in both PCOS ovarian tissues and normal ovarian tissues. The level of SET protein expression in polycystic ovaries was triple higher than that in normal ovaries (*P* < 0.05). *Conclusion*. SET was overexpressed in polycystic ovaries more than that in normal ovaries. Combined with its localization in theca cells, SET may participate in regulating ovarian androgen biosynthesis and the pathophysiology of hyperandrogenism in PCOS.

## 1. Introduction

PCOS is one of the most common reproductive endocrine disorders, which affects approximately 5–10% of premenopausal women and accounts for 75% of anovulatory infertility [1, 2]. PCOS is characterized by hyperandrogenism, chronic anovulation, and polycystic ovaries [1]. It is associated with elevated levels of circulating free testosterone, which is produced primarily in the ovary [3, 4]. In women with PCOS, ovarian theca cells are recognized as one of the primary sources of excessive androgen biosynthesis, which is associated with augmented expression of several steroidogenic enzymes, such as cytochrome P450c17 which has both 17*α*-hydroxylase and C17, 20 lyase activities [5, 6]. In addition to P450c17, the upregulated enzymes also include cytochrome P450 cholesterol side chain cleavage (P450scc), encoded by the *CYP11A *gene [3]. Although some collective studies have identified important correlations between increased steroidogenic enzyme gene expression and increased androgen biosynthesis in PCOS theca cells [7, 8], the exact molecular and cellular mechanisms remain unclear. 

Our previous studies assessed the differential gene expression in human normal and polycystic ovaries using cDNA microarray technology, which identified many genes expressed differentially in PCOS [8]. SET (patient “SE translocation”) was one of the altered genes, which was up-regulated in polycystic ovaries. It was originally identified as a translocated gene in acute undifferentiated leukemia [9]. The SET protein, also known as TAF-1*β* [10–12], I2PP2A [13], and INHAT [14], belongs to a family of multitasking proteins involved in apoptosis, transcription, nucleosome assembly, and histone binding. We did not know what role(s) SET played in the regulation of ovarian steroidogenesis and whether SET would make an attractive candidate for involvement in the hyperandrogenism of PCOS. 

In the present study, we firstly investigated the cellular localization of SET protein in human ovaries and its different expression in the polycystic ovaries compared with human normal ovaries of adult women. It would provide us with preliminary knowledge in the regulation of androgen production in ovary and a fundamental mechanism in the pathophysiology of hyperandrogenism in PCOS.

## 2. Materials and Methods

### 2.1. Ovary Samples and Ethics

Ovarian tissues, samples of normal adult ovaries and polycystic ovaries, were collected during transsexual operation and surgical treatment with the signed consent form. PCOS patients were recruited by the revised criteria of the European Society of Human Reproduction and Embryology/American Society for Reproductive Medicine in 2003: (I) oligomenorrhoea and/or anovulation (eight or fewer menstrual cycles in a year or menstrual cycles more than 35 days in length); (II) clinical and/or biochemical signs of hyperandrogenism; and (III) polycystic ovaries on ultrasound (presence of 12 or more follicles in each ovary measuring 2–9 mm in diameter and/or increased ovarian volume >10 mL) and exclusion of other aetiologies (e.g., congenital adrenal hyperplasia, androgen-secreting tumours, and Cushing's syndrome). The patients did not receive hormonal therapy or stopped it for at least 3 months before operation.

Controls were healthy women, with regular menstrual cycles and without family history of hirsutism. These women had no evidence of hirsutism, acne, or endocrine dysfunction. The hormonal treatment was withdrawn at least 3 months before operation.

The ages of the PCOS specimens group were matched to those of the control group. All samples were histologically examined and stored in liquid nitrogen for Western blotting and paraffin-embedded for immunohistochemical staining. This study was approved by the Institutional Ethics Committee of The First Affiliated Hospital of Nanjing Medical University.

### 2.2. Immunohistochemistry

The paraffin-embedded ovarian tissues were sectioned (5 *μ*m). The detection of SET protein was carried out by a two-step IHC procedure and the primary polyclonal antibody (Abcam, Cambridge Science Park, Cambridge, UK) was diluted by 1 : 400. Immunohistochemical staining of negative controls was executed simultaneously, of which the primary antibody was the IgG.

### 2.3. Western Blot Analysis

The concentrations of protein extracted from the ovarian tissues were measured by the Bradford method. Equal amounts of protein were loaded and separated by 12% SDS-PAGE and then electroblotted onto Polyvinylidene fluoride membrane (Bio-Rad, Hercules, CA). The membranes were incubated with polyclonal antibodies to SET (1 : 1000, Santa Cruz Biotechnology, Santa Cruz, CA, USA). After washing with TBS three times, the membrane was incubated with HRP-conjugated secondary antibodies and then incubated with *β*-tublin (1 : 2000, ABCAM, Cambridge Science Park, Cambridge, UK). After washing with TBS three times, it was incubated with HRP-conjugated secondary antibodies (1 : 1000; Beijing ZhongShan Biotechnology, China) and examined by enhanced chemiluminescence (Amersham Biosciences, Uppsala, Sweden).

### 2.4. Statistical Analysis

Data were expressed as mean ± SEM from six independent experiments in two groups. One-way ANOVA was used for statistical comparison. Values were determined to be significant when *P* ≤ 0.05.

## 3. Results

### 3.1. Cellular Localization of SET Protein in Human Ovaries

**Figure 1:**
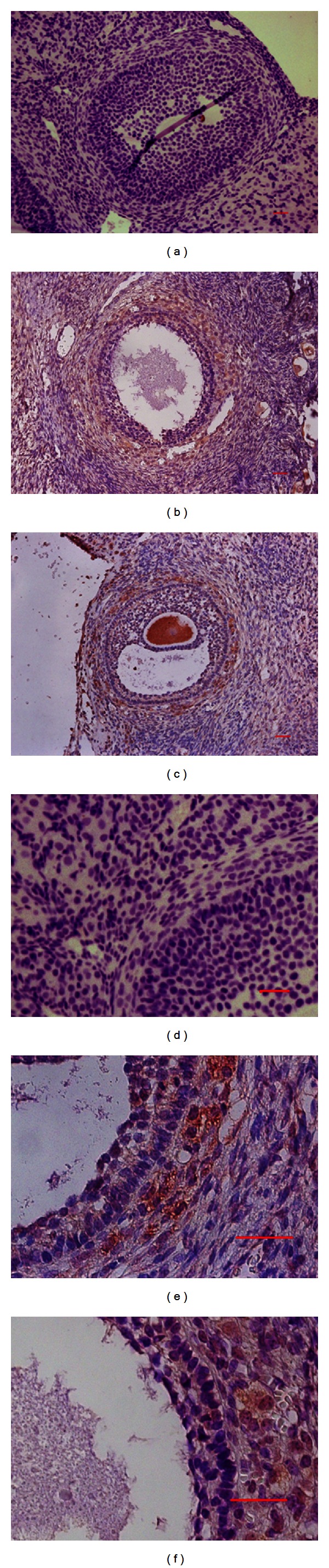
Cellular localization of SET protein in normal and polycystic ovaries. Immunohistochemical localization of SET protein was mainly in the theca cells and oocytes in both PCOS ovarian tissues (b, e) and normal ovarian tissues (c, f). The positive immunoreactive signals were visualized as brown stain ((b, c): original magnification of 20x; (e, f): original magnification of 40x; scale bar = 20 *μ*m). Negative controls were executed simultaneously, of which the primary antibody was the IgG (a, d).

In our study, all PCOS patients, aged from 26 to 31, had menstrual-cycle abnormalities (amenorrhea or oligomenorrhea), polycystic ovaries, and hyperandrogenism (presence of hirsutism and/or serum total T levels >2.43 nmol/L). The control women, aged from 27 to 29, were in good health. To determine the cellular localization of SET protein in the human ovaries, immunohistochemical analysis was performed. As indicated by the results (Figure 1), SET was expressed predominantly in theca cells and oocytes in both PCOS ovarian tissues (Figures 1(b) and 1(e)) and normal ovarian tissues (Figures 1(c) and 1(f)). No signal was detected in the negative control (Figures 1(a) and 1(d)).

### 3.2. Expression of SET Protein in Polycystic Ovaries

**Figure 2:**
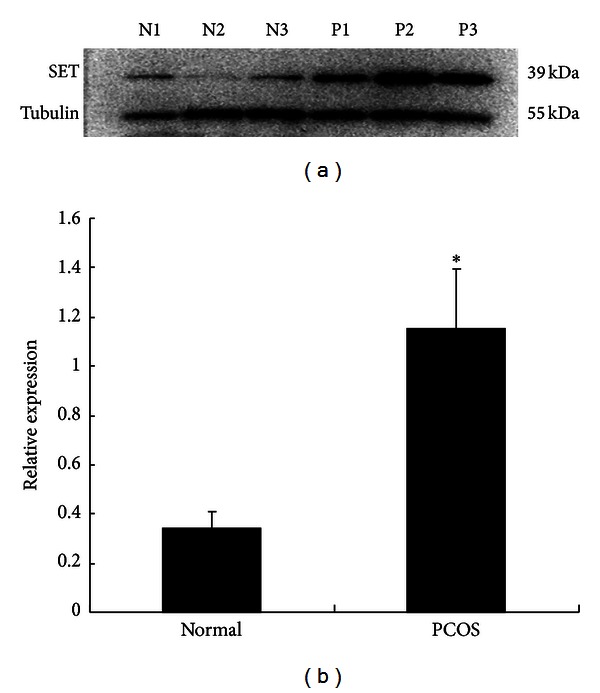
Expression of SET protein in polycystic and normal ovaries by Western blot assay. SET protein of polycystic ovaries was significantly increased when compared with normal ovaries (*P* < 0.05). Tubulin was used as internal controls.

The different expression of SET protein in polycystic ovaries and normal ovaries was detected by Western blot. The protein samples were extracted from six ovaries of normal subjects and PCOS patients. Tubulin was used as internal control. As shown in Figure 2, expression of SET protein in polycystic ovaries was triple times higher than that in normal ovaries (*P* < 0.05).

## 4. Discussion

Androgen biosynthesis in the ovarian theca cells is mediated by steroidogenic enzymes. It is well known that theca cells express P450scc, 3*β*-hydroxysteroid dehydrogenase (HSD3B2), and P450c17 which has both 17*α*-hydroxylase and C17, 20 lyase activities. All of these are key enzymes for androgen synthesis. We found that SET protein was predominantly expressed in theca cells and oocytes of human ovarian tissues (Figure 1). This was in agreement with previous studies performed by Zhang et al. [15] which showed that SET was expressed in theca cells and oocytes of rats. Since the primary function of theca cells is to produce androgen, SET expression in theca cells indicates that SET may be involved in androgen biosynthesis.

SET was originally identified as a translocated gene in acute undifferentiated leukemia [9]. It is a 39 kDa phosphoprotein widely expressed in various tissues, especially in steroidogenic cells within the central nervous system, adrenal gland, and gonad [11, 16]. As a transcriptional regulating factor, SET not only exerts function by binding to the transcriptional coactivators CBP/p300 [12], but also acts directly as a transcriptional factor of P450c17 [16]. SET-mediated promoter hypoacetylation is a prerequisite for coactivation of the estrogen-responsive pS2 gene by PRMT1 [17]. Recent evidence suggested that SET was essential for regulating both the promoter activity of CYP17 and the biological activity of P450c17. In human NT2 neuronal precursor cells and MA-10 cells, SET binds to rat P450c17 promoter at −418/−399 and activates its basal transcription from a rat P450c17 luciferase reporter plasmid [15, 18]. SET has been identified as a potent inhibitor of the protein phosphatase 2A (I2PP2A). SET inhibited PP2A activity by binding to the catalytic subunit PP2Ac through the amino terminal domain (I2NTF; aa 1–175) and the carboxy terminal domain (I2CTF; aa 176–277) [19]. SET can foster 17, 20 lyase activity of P450c17 by inhibiting the dephosphorylation action of PP2A on P450c17 in NCI-H295A cells [20]. All these indicated that SET regulated androgen synthesis in steroidogenic cells by regulation of both the transcriptional and posttranslational levels of P450c17 and CYP17. In mouse eggs PP2A was needed for both continued metaphase arrest and successful exit from meiosis [21], which suggested that SET may participate in oocyte maturation. However, the function of SET protein in human ovary in regulating androgen production and oocyte development should be further studied.

Hyperandrogenism is the central defect in PCOS patients [22], which is related to the increased expressions of steroidogenic enzymes and the increased androgen biosynthesis [23, 24]. Our previous study showed that SET protein was differently expressed in polycystic ovaries by microarray assay [8]. In this study, it is found that SET protein was predominantly expressed in theca cells and oocytes of human ovarian tissues by immunohistochemistry (Figure 1). And expression of SET protein in polycystic ovaries was found to be triple times higher than that in normal ovaries by Western blotting (Figure 2). Combined with the effect of SET on CYP17/P450c17, SET may be involved in the regulation of androgen production in the theca cells, and the elevated SET protein in polycystic ovaries could increase the level of CYP17 and the activity of P450c17, which led partly to the increased production of androgen in theca cells. Additional studies are required to examine whether and how SET overexpression in theca cells contributes to hyperandrogenism of PCOS.

In summary, SET protein expressed predominantly in theca cells, which suggested that SET protein may play a role in modulating androgen production in follicular theca cells. The increased expression of SET protein in polycystic ovarian tissues may be partly a potential mechanism in the pathophysiology of hyperandrogenism in PCOS.
